# Lifestyle Behavior Patterns and Their Association with Active Commuting to School Among Spanish Adolescents: A Cluster Analysis

**DOI:** 10.3390/healthcare13141662

**Published:** 2025-07-10

**Authors:** Pablo Campos-Garzón, Romina Gisele Saucedo-Araujo, Javier Rodrigo-Sanjoaquín, Ximena Palma-Leal, Francisco Javier Huertas-Delgado, Palma Chillón

**Affiliations:** 1 Faculty of Health Sciences, University of Lethbridge, Lethbridge, AB T1K 3M4, Canada; 2 Department of Global Public Health, Karolinska Institutet, 171 77 Stockholm, Sweden; 3 Department of Specific Didactics, Faculty of Education, University of La Laguna, 38200 San Cristóbal de La Laguna, Spain; rsaucedo@ull.edu.es; 4 Sport and Health University Research Institute (iMUDS), University of Granada, 18071 Granada, Spain; 5 Département STAPS, Université de Pau et des Pays de l’Adour, 65000 Tarbes, France; javier.rodrigo@unil.ch; 6 iGEO Group, School of Physical Education, Pontificia Universidad Católica de Valparaíso, Valparaíso 2340025, Chile; ximena.palma.l@pucv.cl; 7 “La Inmaculada” Teacher Training Centre, University of Granada, 18013 Granada, Spain; fjhuertas@ugr.es; 8 Department of Physical Education and Sports, Faculty of Sport Sciences, Sport and Health University Research Institute (iMUDS), University of Granada, 18011 Granada, Spain

**Keywords:** physical activity, sedentary time, screen time, sleep duration, breakfast, transport

## Abstract

Objectives: We aimed to identify clustering patterns of the device-measured physical activity (PA) levels (i.e., light PA and moderate-to-vigorous PA) and sedentary time (ST), screen time, sleep duration, and breakfast consumption of Spanish adolescents and their associations with the mode of commuting to and from schools (i.e., active and passive). Methods: A total of 151 adolescents aged 14.4 ± 0.6 years (53.64% girls) were included in this study. Participants wore an accelerometer device during seven consecutive days to measure PA levels and ST levels. Screen time, sleep duration, breakfast consumption, and the mode of commuting to and from school were self-reported by the participants. A two-step cluster analysis was performed to examine the different lifestyle behavior patterns (defined as data-driven groupings of daily behaviors identified through cluster analysis). Logistic regression models were used to determine the associations among the lifestyle behavior patterns and the mode of commuting to and from school. Results: The main characteristics of the three identified clusters were as follows: (active) high PA levels and low ST (38.4%); (inactive) high sleep duration and daily breakfast consumption, but low PA levels and high ST and screen time (37.2%); and (unhealthy) low PA levels and sleep duration, high ST and screen time, and usually skip breakfast (24.4%). No associations were found between these clusters and the mode of commuting to and from school (all, *p* > 0.05). Conclusions: Three different lifestyle behavior patterns were identified among Spanish adolescents, but no associations were found between these patterns and their mode of commuting to and from school.

## 1. Introduction

Adolescence is a critical phase in human development [1]. During this period, behaviors related to lifestyle, such as physical activity (PA), eating habits, recreational screen time, and sleep patterns, become consolidated [2]. These behaviors tend to persist into adulthood and influence long-term health outcomes [3,4,5]. Various studies have shown that these behaviors do not occur in isolation; rather, they tend to cluster into specific behavioral patterns that interact with each other [6]. Person-centered techniques such as cluster analysis allow researchers to uncover naturally occurring combinations of behaviors that would remain hidden in variable-centered models [7]. By grouping participants rather than single exposures, cluster analysis captures the synergistic and antagonistic interactions among behaviors, offering a fuller picture of real-world lifestyles [7,8]. Nevertheless, despite the well-known benefits, most adolescents do not adopt a healthy lifestyle. Globally, 81% of adolescents do not meet the minimum recommended levels of PA [9]. In Spain, only 1.7% of adolescents simultaneously meet the recommendations for PA, screen time, and sleep [10]. The 24-Hour Movement Guidelines framework suggests integrating these behaviors as interdependent components of a healthy lifestyle [11]. Adhering to these guidelines together seems to generate a synergistic effect that enhances the overall health benefits for adolescents [12]. Furthermore, adopting a healthy behavior, such as PA, can serve as a gateway to others, like healthy eating [13].

A recent systematic review by de Mello et al. [7] highlights that clusters integrating PA, sedentary behavior, sleep, and diet show consistent associations with health indicators in youth and suggests that understanding these combinations is key to designing effective preventive strategies. Furthermore, Leech et al. [14] emphasize the importance of considering not only the combination of behaviors but also their temporal patterns throughout the day to better understand their impact on health. In the Spanish context, the ANIBES study [15] also identified lifestyle clusters among children and adolescents, integrating dietary patterns with PA, sedentary behavior, and sleep. Their findings showed a higher prevalence of obesity in the unhealthy pattern, especially among adolescents from lower socioeconomic backgrounds. Similarly, Sanz-Martín et al. [16] identified, through cluster analysis, three profiles of Spanish adolescents based on their levels of moderate-to-vigorous PA (MVPA), screen time, and sleep duration. Despite the growing interest in analyzing integrated patterns of PA, sedentary behavior, and sleep, there is a limited number of studies that simultaneously include a specific dietary behavior such as breakfast consumption. Its inclusion is particularly relevant because, as a concrete, daily behavior that is sensitive to morning routines, it allows for a more precise capture of how movement behaviors interact throughout the day [14]. Moreover, breakfast consumption has been associated with a lower cardiometabolic risk [17] and better dietary quality [18].

In this scenario, active commuting has long been identified as a key component of healthy lifestyles, and analyzing these behaviors together could provide valuable insights for developing more integrated intervention strategies [19]. Moreover, clustering behaviors can help further explore the relationship between active commuting to and from school (ACS) and other healthy behaviors. It is well established that ACS, particularly walking and cycling, is associated with higher levels of PA [20], reduced sedentary time (ST) [21], and the likelihood that adolescents who engage in ACS are also more active across other time and space domains [22,23]. However, the literature shows inconsistencies in its relationship with other healthy behaviors, including screen time [24], sleep duration [25], breakfast consumption [26], and 24 h movement guidelines [27]. A key reason for these discrepancies is the wide variability in the behaviors themselves. For example, reported home-to-school distance spans from 1.28 ± 0.90 km in Spanish adolescents (active commuters ≈ 0.9 km; passive commuters ≈ 2.0 km) [24] to 3.56 ± 3.28 km in Ecuadorian youth [25]. Other studies categorize commuting only by travel time (<15, 15–30, 30–60, or >60 min) without converting to kilometers [26], while some control for distance analytically but provide no descriptive statistics [27]. Furthermore, most studies have focused on the association between commuting to school, without considering commuting from school, which has been shown to offer distinct benefits, particularly in terms of PA levels [28].

This study is based on the premise that adolescents who maintain a healthy lifestyle, characterized by adequate levels of PA, sufficient sleep, low screen time, and daily breakfast consumption, may be more likely to engage in ACS. Understanding these integrated profiles is therefore crucial for the development of ACS-promotion programmed because reinforcing one healthy habit can serve as an indirect pathway to support active mobility in students’ daily routines. Furthermore, exploring how data-driven behavioral patterns relate to both legs of the school journey can guide public health interventions that target multiple behaviors simultaneously. Although clustering PA, ST, screen time, sleep duration, and breakfast consumption has been linked to significant health outcomes [29,30], no previous study has combined device-measured movement behaviors with dietary and screen-based routines to generate lifestyle clusters and examine their association with both to and from school commuting in Spanish adolescents. By applying a two-step cluster analysis, well suited to mixed continuous and categorical inputs, we aim to capture naturally occurring combinations of behaviors that variable-centered approaches may overlook. Accordingly, this study sought to identify clustering patterns of device-measured PA levels (i.e., light PA [LPA] and MVPA), ST, screen time, sleep duration, and daily breakfast consumption among Spanish adolescents and to explore their associations with modes of commuting to and from school.

## 2. Materials and Methods

The current cross-sectional study was conducted under the umbrella of the PACO (Walking and Cycling to School). Briefly, the PACO study aimed to promote cycling to and from school among Spanish adolescents through a school-based intervention. Regarding the school recruitment process, all secondary schools from four Spanish cities were invited to participate in the PACO study via phone calls. From those who agreed to participate, 12 schools in four Spanish cities (Almería, Granada, Jaén, and Valencia) were randomly selected. For the subsequent recruitment of participants, the research team requested a meeting with the Physical Education teacher to explain the objectives of the study and the data collection process. During this meeting, informed consent forms were provided for the adolescents to take home. These forms needed to be signed by their parents or legal guardians if they accepted to participate in the study. More information about the sample size justification and recruitment process can be found elsewhere [31]. The PACO study was approved by the Review Committee for Research Involving Human Subjects at the University of Granada (Reference: 162/CEIH/2016).

### 2.1. Participants

A total of 288 participants, aged between 13 and 16 years, from the 12 schools returned informed consent forms signed by their parents or legal guardians and wore an accelerometer attached to their hip. Participants were included in the analysis if they met the following criteria: (1) provided valid accelerometer data, defined as at least three weekdays and one weekend day with a minimum of eight hours of wear time per day; (2) provided complete sociodemographic (i.e., age, gender, and postal address) and socioeconomic status data; and (3) provided valid self-reported data on screen time, sleep duration, daily breakfast consumption, and the mode of commuting to and from school. Of the initial sample, 100 participants were excluded due to insufficient accelerometer data, and 37 were excluded for missing data on commuting mode. As a result, a total of 151 adolescents (mean age = 14.4 ± 0.6 years) were included in the final statistical analyses.

### 2.2. Procedures

The data collection process took place from January 2019 to June 2021. Data were collected during two separate visits to the secondary schools. During the first visit, participants were given an accelerometer and instructed on how to use it. They had to wear it on a belt around their waist for seven consecutive days and remove it only for sleep and water-based activities. The second visit occurred eight days after the first, during which the accelerometers were collected, and participants completed a paper-based questionnaire where they self-reported their sociodemographic data, family socioeconomic data, sleep duration, daily breakfast consumption, and the mode of commuting to and from school. Afterward, using the participants’ self-reported postal addresses, the distance from home to school was calculated, as well as the walkability index within a 1350 m buffer around the self-reported address.

### 2.3. Measures

#### 2.3.1. Sociodemographic Data and Family Socioeconomic Status

Participants self-reported their sociodemographic data (i.e., gender, age, and postal address) via a paper-based questionnaire. Family socioeconomic status was assessed using an adaptation of the family affluence scale (FAS) [32]. Participants were asked about the number of computers (0: none, 1: one, 2: two, or 3: three or more) and four-wheeled vehicles in their household (0: none, 1: one, or 2: two or more). The final FAS score was the average of these questions, ranging from 0 (not having) to 4 (having two or more computers and vehicles).

#### 2.3.2. Sedentary Time and PA Levels

Sedentary time and PA levels were measured using a triaxial accelerometer (Actigraph GT3x+, Pensacola, FL, USA). Actilife software (Actigraph, v.6, Pensacola, FL, USA) was used for device initialization and data downloading. Accelerometers were programmed with a frequency of 90 Hz. For accelerometer data processing, the open-source R package GGIR v.3.0-0 [33] was used (https://cran.r-project.org/web/packages/GGIR/index.html) (accessed on 12 March 2025). The GGIR pipeline included the following: (1) the auto-calibration of raw accelerometer data based on local gravitational acceleration [33]; (2) the detection and imputation of non-wear time [34]; (3) the calculation of activity counts over 15 s epochs using the algorithm described by Neishabouri et al. [35], facilitated by the open-source R package actilifecounts v.1.1.1 (https://cran.r-project.org/web/packages/actilifecounts/index.html) (accessed on 12 March 2025); and (4) the classification of sedentary time as 0–26 counts/15 s, LPA as 26–574 counts/15 s, and MVPA as 574 counts/15 s [36].

#### 2.3.3. Screen Time

Adolescents self-reported their screen time from the Youth Activity Profile questionnaire developed by Saint-Maurice & Welk [37] and validated in Spanish adolescents [38]. The items related to screen time were “How much time did you spend watching television outside of school?”, “How much time did you spend playing video games outside of school?”, “How much time did you spend using the computer outside of school?”, and “How much time did you spend using your mobile phone outside of school?”. The response options for all the questions were as follows: (a) I never used it; (b) I used it for less than 1 h per day; (c) I used it for 1–2 h per day; (d) I used it for more than 2 h and up to 3 h per day; and (e) I used it for more than 3 h per day. The participants’ responses were used to calculate the number of hours they spent using screens: “never used it” was categorized as “0 h”, “less than 1 h per day” was categorized as “0.5 h”, “1–2 h per day” was categorized as “1.5 h”, “more than 2 h and up to 3 h” was categorized as “2.5 h”, and “more than 3 h per day” was categorized as “3 h”.

#### 2.3.4. Sleep Duration and Breakfast Consumption

Sleep duration and daily breakfast consumption were assessed by validated questionnaires [24,25]. Participants self-reported the exact time in hours and minutes when they went to sleep and when they woke up using the following questions: “What time do you go to sleep every day?” and “What time do you wake up every day?”. The research staff calculated the number of minutes between the time students went to sleep and the time they woke up to determine each student’s sleep duration. Regarding daily breakfast consumption, participants were asked how many days they had breakfast using the following question: “From Monday to Friday during the weeks you go to school, how many days do you usually have breakfast?”. The response options were “I never have breakfast on school days”, “1 day”, “2 days”, “3 days”, “4 days”, or “5 days”. A continuous variable from 0 to 5 was created based on participants’ responses.

#### 2.3.5. Mode of Commuting to and from School

Participants self-reported their usual mode of commuting to and from school through the following questions from a reliable questionnaire [39]: “How do you usually commute to school?” and “How do you usually commute from school?”. In both cases, the response options were “I didn’t go to school”, “walking”, “cycling”, “car”, “motorbike”, “school bus”, “public bus”, “train/metro”, and “other”. Participants who reported walking or cycling were categorized as engaging in “Active commuting to school” and “Active commuting from school”, while those who reported using a car, motorbike, school bus, public bus, or train/metro were categorized as engaging in “Passive commuting to school” or “Passive commuting from school”. Other modes of commuting were categorized as “active commuting to school” or “active commuting from school” if, for example, they involved a kick scooter; otherwise, they were categorized as “passive commuting to school” or “passive commuting from school”.

#### 2.3.6. Environment Characteristics

For both the distance from home to school and the home walkability index, ArcGIS 10.3 software (ESRI, Redlands, CA, USA) was used. The shortest route between each participant’s home and school was used to determine the distance from home to school. Additionally, the walkability index around the postal addresses self-reported by participants was calculated. The home walkability index included three built-environment characteristics previously associated with PA levels [40,41]: (1) net residential density, calculated by dividing the number of residential units in an area by the amount of land designated for residential use; (2) land use mix, which captured the distribution of different land use types (e.g., office or recreational); and (3) intersection density, calculated by dividing the number of street intersections in a block group by the total land area, excluding freeways and inaccessible roads. Therefore, the home walkability index was calculated as [(z-score of intersection density) + (z-score of net residential density)] [42].

### 2.4. Statistical Analysis

The descriptive characteristics of the sample were reported as means and standard deviations (continuous variables) or frequencies (categorical variables). The differences between boys and girls were calculated using the independent *t*-test (continuous variables) or the chi-square test (categorical variables).

The cluster analysis was implemented in two main phases. The first one consisted of a principal component analysis (PCA) including all variables of the study. The second one performed a hierarchical cluster analysis with the PCA generated in the previous analysis. To identify underlying patterns between measurements considered all together, a PCA was performed with several variables; some are used as active ones (age, ST, LPA, MVPA, all screen time variables, sleep duration, and daily breakfast consumption), and another one as a supplementary variable (gender). The selected variables were included based on prior evidence linking each of them individually to active commuting to school, with the aim of capturing integrated lifestyle patterns most relevant to commuting behaviors in adolescents [24,25,26,27]. The results are displayed on a graph denoted as the first principal plane available for the variables (the correlation circle) constructed using the biggest two dimensions, also called principal components (PCs). This method allows us to identify correlations between variables and to extract characteristics revealing heterogeneity between subjects; each dimension is interpreted in this way using the contributions of each variable.

The cluster hierarchical analysis using Euclidean distance and Ward aggregation criterion was performed on the five PCs as an exploratory tool to uncover natural clusters within the dataset. The best number of clusters to group all participants was decided automatically by the hierarchical analysis. The results are displayed using “ellipse plots” with different clusters of participants on the first correlation circle. Several clusters were retained among subjects and characterized in terms of health behaviors variables, giving several typologies of the participants. The optimal cluster solution was determined with NbClust, inspecting the elbow plot, average silhouette width, Hubert–L index and gap statistic; a three-cluster solution (average silhouette = 0.36) was retained. Finally, two logistic regression models (one for each trip direction) were carried out to examine the association between the mode of commuting to and from school (dependent variables) and the different lifestyle behavior patterns identified in the cluster analysis (independent variables). All the models were adjusted by gender, the FAS, distance from home to school, and the home walkability index.

All analyses and graphics were performed using R v.4.2.1 (R Foundation for Statistical Computing, Vienna, Austria). PCA and hierarchical clustering on principal components were performed using the R package FactoMiner v.2.11, and FactoExtra v.1.07 was used to construct the graphs. The significance level was set at *p* < 0.05 to establish statistical significance.

## 3. Results

### 3.1. Participants’ Characteristics

Table 1 provides a general description of the participants and outcomes measured in this study. The sample size was 151 participants (53.64% girls) aged 14.32 (±0.59) years. Girls accumulated significantly higher ST and smartphone time than boys (577.31 min vs. 606.83 min, *p* = 0.041; 2.20 h vs. 2.64 h, *p* = 0.043, respectively) but significantly lower time playing videogames and total screen time than boys (1.17 h vs. 0.26 h, *p* < 0.001; 4.50 h vs. 5.30 h, *p* = 0.047, respectively). Girls lived in lower walkable environments than boys (2.17 vs. 1.66, *p* = 0.003). Moreover, girls had a significantly lower percentage of meeting the sleep guidelines than boys (32.0% vs. 24.0%, *p* = 0.041).

### 3.2. Principal Component Analysis

Five major dimensions were identified accounting for more than 70% of the variability. The two main dimensions or PCs described correlation and inverse correlation between groups of variables. PC1 was integrated by screen time behaviors, while MVPA and LPA mainly contributed to PC2 (see Supplementary Figure S1). Also, PCA correlations indicated that participants with higher values of the first group of variables will score lower in the second group and vice versa, showing inverse correlation between these variables. All PCA data are available in Supplementary Table S1.

### 3.3. Clusters

**Figure 1:**
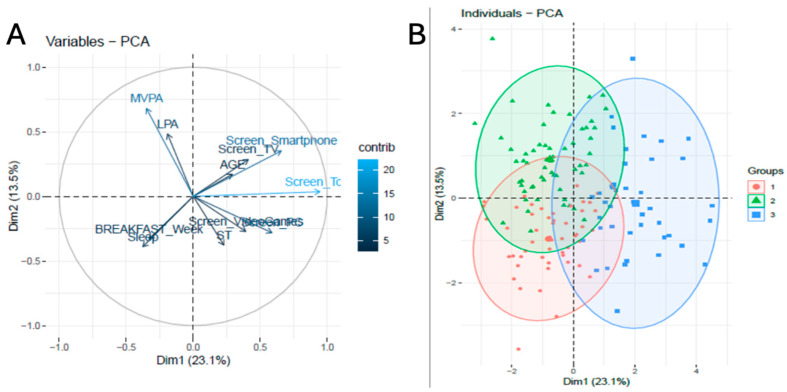
Principal component analysis (PCA) correlation circle (**A**) and cluster classification based on PCA (**B**). Notes: In panel (**A**), arrows represent the correlation between the original variables and the principal components (Dim1 and Dim2), where the direction indicates the association with each component and the length reflects the strength of the contribution. Dim1 (23.1%) and Dim2 (13.5%) represent the percentage of variance explained by the first and second principal components, respectively. Panel (**B**) displays the distribution of individuals in the PCA space, colored by cluster membership. Each point represents one participant, and ellipses denote the 95% confidence region for each cluster (Group 1: red, Group 2: green, and Group 3: blue).

The cluster analysis is visually represented in Figure 1, showing the correlation circle and how variables are grouped based on the two main dimensions of PCA (i.e., PC1, PC2). Also, this figure illustrates the clusters of participants based on the same axes, allowing us to identify which clusters are more linked with each group of variables. In this sense, cluster 1 is mainly composed of higher values of sleep duration, daily breakfast consumption, and ST, cluster 2 is mainly composed by higher values of LPA and MVPA, an important representation of sleep duration, and daily breakfast consumption, and cluster 3 is integrated by sedentary behaviors, a lack of sleep, and skipping daily breakfast. The vectors represented in the correlation circle describing LPA and MVPA are positively correlated between them and inversely correlated with ST, videogames, and computer time. On the other axis, the vectors show that sleep duration and daily breakfast consumption are positively correlated between them while inversely correlated with age, TV time, smartphone time, and total screen time.

**Figure 2:**
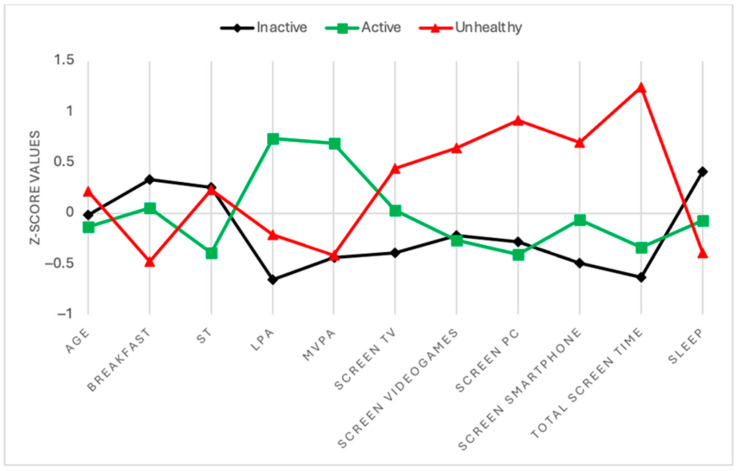
Cluster representation based on standardized values (z-scores). Notes: Each line represents the standardized profile (z-scores) of one of the identified clusters across all included behavioral and demographic variables.

The clustering hierarchical algorithm identified three clusters of participants and Table 2 describes the most important characteristics of them accounting for each cluster. First, cluster 1 was named “inactive lifestyle” as it is mainly composed by participants scoring the highest values of sleep duration and daily breakfast consumption, but they had the lowest scores on PA levels (i.e., LPA, MVPA) and the highest ST. Second, cluster 2 was named “active lifestyle” because participants scored the highest values of LPA and MVPA and the lowest values of ST, computer time, and videogame time. Thirdly, cluster 3 was named “unhealthy” as it includes participants with the highest values of sedentary behaviors, the lowest of sleep duration and daily breakfast consumption, and low values of MVPA and LPA. The cluster analysis revealed there was no significant difference between clusters by the gender variable. Also, after performing a one-way ANOVA, age was the only variable without significant differences between clusters. In summary, all differences between variables among the three clusters are represented with standardized values (i.e., z-scores) in Figure 2.

### 3.4. Regression Models

The association between the mode of commuting to and from school and the different lifestyle behavior patterns is presented in Table 3. When compared to the reference group (unhealthy lifestyle), adolescents in the active lifestyle cluster showed non-significant higher odds of actively commuting to school (OR = 1.20, 95% CI: 0.50–2.89; *p* = 0.68) and from school (OR = 1.39, 95% CI: 0.56–3.46; *p* = 0.53). Similarly, those in the inactive lifestyle cluster also showed non-significant associations for commuting to school (OR = 1.23, 95% CI: 0.52–2.94; *p* = 0.66) and from school (OR = 1.23, 95% CI: 0.50–3.07; *p* = 0.67).

## 4. Discussion

### 4.1. Main Findings

This study aimed to identify clustering patterns of the device-measured PA levels (i.e., LPA and MVPA), ST, screen time, sleep duration, and daily breakfast consumption of Spanish adolescents and their associations with the mode of commuting to and from schools. Our results suggest that three different lifestyle behaviors patterns among Spanish adolescents were identified and characterized as follows: (1) Active lifestyle: high LPA and MVPA levels, low ST, low total screen time, medium sleep time, and daily breakfast almost every day. (2) Inactive lifestyle: low LPA and MVPA levels, high ST but very low screen time, high sleep duration, and daily breakfast. (3) Unhealthy lifestyle: low LPA and MVPA levels, high sedentary behaviors, low sleep duration, and usually skip breakfast. However, no associations were found between the mode of commuting to and from school and the different lifestyle behaviors established.

### 4.2. Behavioral and Practical Significance of Identified Lifestyle Patterns

**Figure 3:**
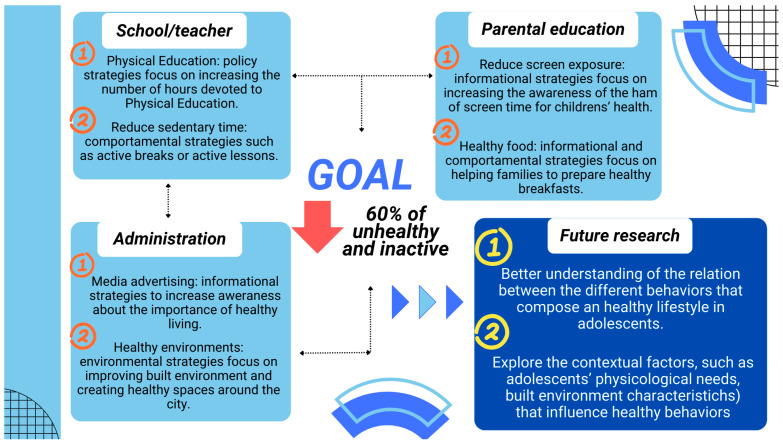
Practical recommendations and future directions based on the cluster analysis results.

In the current study, three different behavior patterns were identified: active, inactive, and unhealthy lifestyles. In the scientific literature the active and unhealthy lifestyles are the most commonly identified [43,44]. Indeed, these results are quite common between high-income countries [43,45]. Our findings align with previous studies that reported the most active adolescents tend to accumulate less ST and higher PA levels [46]. Similar “high-PA/low-SB” clusters have consistently been linked to favorable adiposity, cardiometabolic and mental health outcomes among European adolescents [7]. From an intervention standpoint, these adolescents mainly require strategies that help maintain their balanced routine (e.g., ensuring neighborhood and school environments remain supportive of active commuting options throughout upper-secondary transitions). This profile is not surprising, given that the literature suggests that adopting healthy behaviors often leads to adapting more healthy behaviors [12]. Other studies have similarly identified groups with low PA levels and high sedentary behaviors, which are also associated with poor diet quality [47,48,49], possibly due to the excessive screen time contributing to these outcomes (i.e., more screen time more exposure to fast-food advertisements) [50]. In contrast, inactive lifestyle, despite having an adequate sleep duration, daily breakfast consumption and low screen time, stands out for its high ST and low PA levels, adding further controversy to the scientific literature, as studies have typically identified clusters with either high screen time and high PA [51,52] or high screen time and low PA [43,53]. Clusters with adequate sleep but deficient movement have been reported in Portuguese and Norwegian samples and predict poorer cardiorespiratory fitness trajectories despite otherwise “healthy” routines [54]. It has been suggested that clustering patterns may be related to the culture of particular countries [45,55], increasing the complexity of behavioral grouping. In practical terms, programs for this group should prioritize sedentary time substitution (standing lessons and active classroom breaks) rather than simply reducing screen media, which is already limited. Finally, the unhealthy lifestyle cluster concentrates multiple risk factors: low PA, elevated sedentary and screen time, short sleep and breakfast skipping. Comparable “sedentary-screen/poor-diet” clusters are associated with higher adiposity, depressive symptoms and pro-inflammatory profiles in cross-national youth cohorts [56]. This pattern reinforces calls for multicomponent interventions that combine screen time limits, parental support for regular breakfast and incentives for active travel. The most concerning aspect of these results is that over 60% of the participants (inactive and unhealthy lifestyles) exhibit worrisome behaviors that need to be addressed. It is indeed a troubling picture, as prolonged exposure to clusters of unhealthy lifestyle habits may be associated with both short- and long-term health risk factors, which are precursors to chronic diseases [57,58]. Therefore, these results underline the need for interventions aimed at promoting healthier lifestyles in adolescents that address these aspects simultaneously (e.g., PA levels, ST, screen time), rather than in isolation, which may be more effective in improving adolescents’ overall health [59]. Future studies should explore the contextual factors (e.g., adolescents’ psychological needs, built environment characteristics) that may influence these behaviors, as well as the evolution of these patterns over time to better understand their long-term impact (see Figure 3).

### 4.3. Associations Between Lifestyle Patterns and School Commuting Behaviors

Despite the clear distinction between the three lifestyle behaviors, no associations were found between them and the mode of commuting to and from school. These results may be surprising, but it highlights the inconsistency in the associations between some healthy behaviors and the mode of commuting to and from school. It is well-known that adolescents who engage in ACS accumulate higher MVPA [23,60], LPA [61], and lower ST [21] compared to those who use other modes of commuting. Regarding screen time, it has been found that those who commute actively to school report higher screen time [62], or there is no significant association between screen time and the mode of commuting to school [24]. Similar results have been found for daily breakfast consumption, where students who eat breakfast were either not engaging in active commuting to school [26] or no associations were observed [24,25]. In terms of sleep duration, more positive findings suggest that adolescents who meet sleep recommendations are more likely to engage in ACS [24,25,63]. Overall, these findings highlight the complexity of the interactions between health behaviors and the mode of commuting to and from school. While PA appears to be strongly linked to ACS, the lack of consistent associations with other behaviors, such as screen time, sleep duration, or breakfast consumption, underscores the need for further research to explore these connections in greater depth. Moreover, it is important to note that ACS is a behavior that may depend not only on the individual, but also on parental influence, environmental characteristics, or barriers of the adolescents themselves [64,65,66]. In fact, several mechanisms may explain this lack of alignment. Built-environment constraints (e.g., sidewalk availability, traffic safety, school distance) can override personal activity profiles when adolescents choose how to commute to and from school [67]. Parental decision-making and safety concerns also modulate school travel mode, sometimes counteracting adolescents’ own preferences [68]. Moreover, behavioral compensation may occur: active cluster adolescents who obtain substantial MVPA through organized sport could perceive less need to walk or cycle to school, whereas unhealthy cluster peers may rely on motorized travel due to morning fatigue linked to late-night screen use and curtailed sleep [69,70]. These multilevel influences highlight that, while ACS can contribute meaningfully to overall PA, its adoption depends on a complex interplay of individual routines, parental autonomy and environmental feasibility. Future longitudinal research should examine how changes in these contextual factors facilitate (or hinder) transitions between clusters and their associated commuting behaviors.

### 4.4. Limitations and Strengths

The current study is not without limitations. Firstly, the cross-sectional design does not allow for cause-and-effect interpretation. Secondly, although the schools were randomly selected, the study sample is not representative of all Spanish adolescents, and the results of this study should not be extrapolated to other geographical areas or age groups. Thirdly, self-reported measures of screen time, sleep duration, and breakfast consumption were used, which could lead to recall bias associated with this type of instrument. Despite these limitations, this study has several important strengths: (1) the use of accelerometry meant we could estimate the ST and PA levels; (2) the application of PCA meant we were able to group similar groups variables and identify five dimensions explaining more than 70% of the data; (3) the hierarchical cluster analysis allowed us to independently categorize the sample into three different groups of participants; (4) both trip directions were considered when establishing associations between the mode of commuting to and from school and lifestyle behavior patterns; (5) and to the best of our knowledge, it is the first study to analyze the associations of different lifestyle behavior patterns jointly with the mode of commuting to and from school.

## 5. Conclusions

Three lifestyle behavior patterns were identified among Spanish adolescents based on PA levels, ST, screen time, sleep duration, and breakfast consumption. These groups were characterized by high PA levels and low sedentary time (active lifestyle), adequate sleep duration and daily breakfast but low PA levels and high sedentary time (inactive lifestyle), and low PA levels and sleep duration, high sedentary time, and frequently skipping breakfast (unhealthy lifestyle). However, no associations were found between these lifestyle behavior patterns and the mode of commuting to and from school.

These findings have significant public health implications, as categorizing adolescents based on lifestyle behavior patterns may help identify appropriate interventions. This, in turn, can contribute to developing healthier youth, which will lead to healthier adults. The differentiated profiles observed here call for equally differentiated actions. For adolescents in the active cluster, the priority is maintenance: municipalities should preserve safe walking and cycling routes around schools, while teachers can reinforce active travel habits by scheduling “walk or wheel” days during key school transitions. Students in the inactive cluster would profit most from classroom strategies that replace sitting with light movement: standing lessons, two-minute activity breaks, or active homework tasks. The unhealthy cluster requires multicomponent programs that couple breakfast clubs and screen time limits with incentives for active commuting (e.g., mileage reward schemes) and traffic-calming infrastructure. Embedding these tailored approaches within whole-school health policies could maximize reach and sustainability. Consequently, understanding to which cluster adolescents belong would allow administrations and schools to correctly develop the most effective strategies in order to increase healthy lifestyles in adolescents.

Longitudinal studies are needed to track how adolescents migrate between clusters over time and to test causal links with commuting modes. Cluster-tailored intervention trials, ideally conducted across diverse geographic and socioeconomic contexts, should examine whether integrated packages (e.g., combining breakfast provision, in-class activity breaks, and safe route initiatives) can shift both lifestyle patterns and travel behavior. Incorporating objective measurements of diet, screen exposure and built-environment features will further clarify mechanisms and strengthen the evidence base for policy.

## Figures and Tables

**Table 1:** Descriptive characteristics of the participants.

	Total Sample (n = 151)	Girls (n = 81)	Boys (n = 70)	*p*
	Mean (SD)	Mean (SD)	Mean (SD)	
Age (year)	14.32 (0.59)	14.25 (0.51)	14.41 (0.66)	0.155
FAS	3.3 (0.84)	3.30 (0.83)	3.28 (0.85)	0.892
ST (min)	593.15 (88.14)	606.83 (83.95)	577.31 (90.78)	**0.041**
LPA (min)	156.99 (40.56)	153.47 (36.25)	161.08 (44.96)	0.259
MVPA (min)	38.75 (19.44)	38.66 (17.55)	38.86 (21.55)	0.951
TV time (min)	0.91 (0.85)	0.91 (0.85)	0.91 (0.86)	0.996
Videogame time (min)	0.68 (0.62)	0.26 (0.21)	1.17 (1.32)	**<0.001**
Computer time (min)	0.83 (0.74)	0.68 (0.49)	1.01 (0.94)	0.098
Smartphone time (min)	2.43 (1.33)	2.64 (1.27)	2.20 (1.37)	**0.043**
Total screen time (min)	4.87 (2.53)	4.50 (2.35)	5.30 (2.69)	**0.047**
Sleep time (hours)	7.81 (1.08)	7.71 (1.11)	7.91 (1.05)	0.227
Daily breakfast consumption (day)	3.86 (1.87)	3.90 (1.81)	3.82 (1.95)	0.814
Active commuters to school (n, %)	97 (64.23%)	45 (55.55%)	42 (60%)	0.050
Active commuters from school (n, %)	100 (66.20%)	60 (74.07%)	40 (57.14%)	**0.046**
Distance from home to school (m)	3649.20 (8376.23)	2692.89 (3128.50)	4463.82 (10,995.24)	0.169
Home walkability index	1.90 (1.42)	1.66 (1.28)	2.17 (1.53)	**0.003**
**24 h Movement Behaviors Guidelines**				
MVPA guidelines (n, %)	24 (16%)	13 (16%)	11 (15.7%)	0.955
Screen time guidelines (n, %)	21 (14%)	14 (17.3%)	7 (10%)	0.197
Sleep time guidelines (n, %)	56 (37%)	24 (29.6%)	32 (45.7%)	**0.041**
24 h guidelines (n, %)	6 (4%)	3 (3.7%)	3 (4.3%)	0.855

Note. Boldfaced *p*-values refer to values ≤ 0.05. Abbreviations: ST = sedentary time; LPA = light physical activity; MVPA = moderate-to-vigorous physical activity; TV = television; FAS = family affluence scale; m = meters; min = minutes; SD = standard deviation; n, % = frequency, percentage.

**Table 2:** Descriptive characteristics of adolescents classified into three lifestyle behavior patterns identified through cluster analysis: inactive, active, and unhealthy lifestyles.

	Inactive Lifestyle (n = 51)	Active Lifestyle (n = 58)	Unhealthy Lifestyle (n = 42)	F	*p*	*ηp* ^2^
	Mean (SD)	Mean (SD)	Mean (SD)			
Age (years)	14.31 (0.64)	14.24 (0.47)	14.45 (0.66)	1.53	0.213	0.01
ST (min)	615.41 (78.61) ^a^	558.81 (77.73) ^a,c^	613.54 (98.87) ^c^	8.46	<0.001	0.06
LPA (min)	130.28 (25.18) ^a,b^	186.87 (32.51) ^a,c^	148.17 (39.32) ^b,c^	52.19	<0.001	0.28
MVPA (min)	30.21 (13.31) ^a^	52.14 (18.81) ^a,c^	30.63 (16.21) ^c^	28.23	<0.001	0.17
TV time (hours)	0.54 (0.43) ^b^	0.94 (0.85)	1.32 (1.18) ^b^	9.84	<0.001	0.07
Videogame time (hours)	0.44 (0.32) ^b^	0.38 (0.26) ^c^	1.40 (1.16) ^b,c^	7.62	<0.001	0.06
Computer time (hours)	0.51 (0.36) ^b^	0.34 (0.22) ^c^	1.92 (1.54) ^b,c^	20.02	<0.001	0.14
Smartphone time (hours)	1.77 (1.23) ^a,b^	2.34 (1.22) ^a,c^	3.36 (1.06) ^b,c^	23.02	<0.001	0.15
Total screen time (hours)	3.26 (1.52) ^a,b^	4.01 (1.43) ^a,c^	8.02 (1.85) ^b,c^	94.79	<0.001	0.40
Sleep time (hours)	8.25 (1.16) ^a,b^	7.73 (0.85) ^a^	7.38 (1.08) ^b^	7.11	0.002	0.05
Daily breakfast consumption (day)	4.49 (1.27) ^b^	3.95 (1.91) ^c^	3.25 (2.12) ^b,c^	7.81	<0.001	0.06

Note: Data are presented as mean (SD). Superscript letters denote statistically significant differences between clusters (*p* < 0.05) according to one-way ANOVA with Bonferroni post hoc tests. Abbreviations: ST = sedentary time; LPA = light physical activity; MVPA = moderate-to-vigorous physical activity; TV = television; SD = standard deviation.

**Table 3:** Associations between the mode of commuting to and from school and the different lifestyle behavior patterns established.

	Unhealthy Lifestyle	Active Lifestyle	Inactive Lifestyle
	Ref.	OR (CI 95%)	OR (CI 95%)
Mode of commuting to school	Ref.	1.20 (0.50, 2.89)	1.23 (0.52, 2.94)
Mode of commuting from school	Ref.	1.39 (0.56, 3.46)	1.23 (0.50, 3.07)

Note: Models adjusted for gender, family affluence scale (FAS), distance from home to school, and home walkability index. Mode of commuting to and from school reference: active commuting. Abbreviations: Ref = reference; CI = confidence interval; OR = Odds ratio.

## Data Availability

The authors have permission to share data. Requests can be sent to the corresponding author.

## Supplementary Materials

The following supporting information can be downloaded at https://www.mdpi.com/article/10.3390/healthcare13141662/s1, Figure S1: Correlations and contributions between targeted variables and Principal Components (dimensions); Table S1: Correlations between Principal Components and study variables.

## Abbreviations

The following abbreviations are used in this manuscript:

PA	Physical activity
ST	Sedentary time
MVPA	Moderate-to-vigorous physical activity
LPA	Light physical activity
ACS	Active commuting to and from school
PCA	Principal component analysis
PCs	Principal components
