# Supplementary material for: Lifestyle Behavior Patterns and Their Association with Active Commuting to School Among Spanish Adolescents: A Cluster Analysis
Source: Healthcare (Basel). 2025 Jul 10;13(14):1662. doi: 10.3390/healthcare13141662 (PMC12294203; doi:10.3390/healthcare13141662)
Supplement: Supplementary file 1 [file healthcare-13-01662-s001.zip › healthcare-3681404-supplementary.pdf]

SUPPLEMENTARY MATERIAL

Supplementary Figure S1. Correlations and contributions between targeted variables and Principal Components (dimensions).

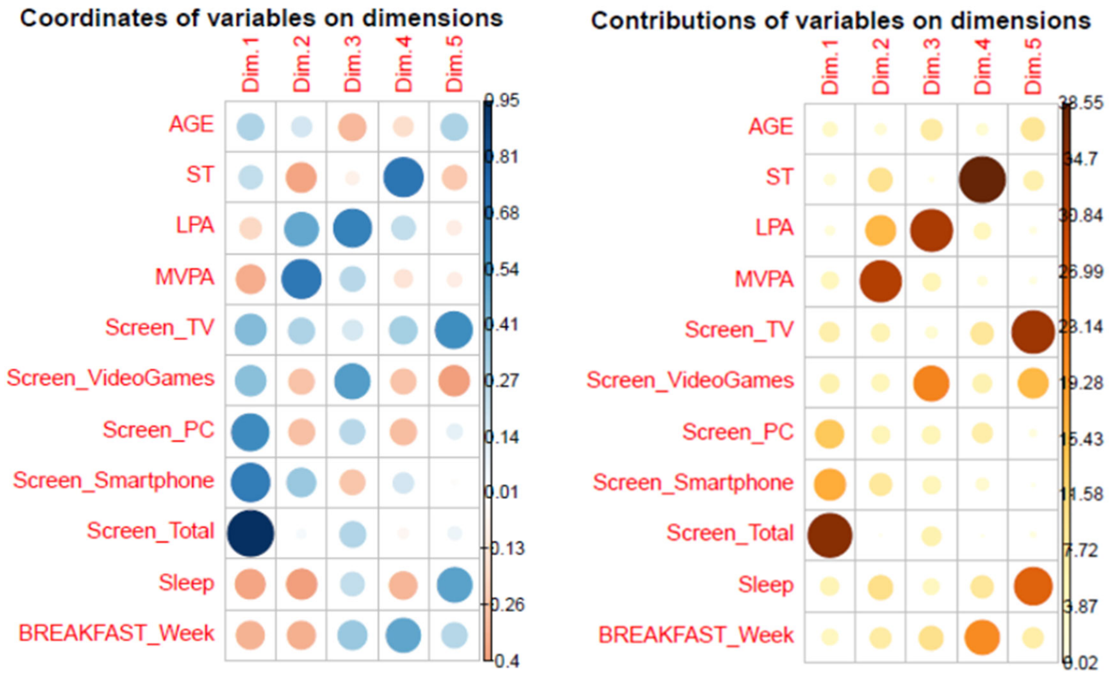

Notes: The gradient of the right-side scale on both images indicate the correlation score and contribution percentage of each variable to each one of the five dimensions.

Supplementary Table S1. Correlations between Principal Components and study variables.

	Principal Components (Dimensions)				
	PC1	PC2	PC3[1]	PC4[1]	PC5
Eingenvalues	2.53	1.48	1.27	1.20	1.01
Quantitative Variables					
Age (yr.)	0.29	0.17	-0.31	-0.16	0.30
Breakfast (day)	-0.32	-0.33	0.35	0.50	0.26
ST (min)	0.22	-0.37	-0.07	0.68	-0.25
LPA (min)	-0.19	0.48	0.63	0.23	-0.08
MVPA (min)	-0.34	0.68	0.25	-0.13	-0.08
TV time (hours)	0.41	0.28	0.16	0.32	0.57
Videogames time (hours)	0.39	-0.27	0.52	-0.26	-0.39
Computer time (hours)	0.58	-0.28	0.26	-0.28	0.09
Smartphone time (hours)	0.65	0.35	-0.25	0.17	-0.01
Total screen time (hours)	0.94	0.03	0.28	-0.04	0.07
Sleep time (hours)	-0.37	-0.38	0.23	-0.32	0.51
Qualitative variable (Gender)					
Boys	0.15	-0.08	0.37	-0.33	-0.02
Girls	-0.13	0.07	-0.32	0.28	0.01

Notes: [1] = $p<0.05$ by Gender. PC = Principal Component.
